# Medical emergencies at sea: an analysis of ambulance-supported and autonomously performed operations by lifeboat crews

**DOI:** 10.1186/s12873-023-00879-7

**Published:** 2023-09-19

**Authors:** Daphne M. Messelink, Gert-Jan van der Ploeg, Theo van der Linden, Roos D. Flameling, Joost J. L. M. Bierens

**Affiliations:** 1 Department of Internal Medicine, Ziekenhuis Groep Twente Hospital, Zilvermeeuw 1, 7609 PP Almelo, The Netherlands; 2 Regional Ambulance Service Noord-Holland Noord, Hertog Aalbrechtweg 22, 1823 DL Alkmaar, The Netherlands; 3 The Royal Dutch Lifeboat Institution (KNRM), Haringkade 2, 1976 CP IJmuiden, The Netherlands; 4 Regional Ambulance Service Ambulance Oost, Demmersweg 55, 7556 BN Hengelo, The Netherlands; 5 Extreme Environments Laboratory, School of Sport, Health & Exercise Science, University of Portsmouth, Portsmouth, UK

**Keywords:** Lifeboat crew, Sea rescue, Ambulance crew, Search and rescue, Dispatch, Telemedicine

## Abstract

**Background:**

Very little data is available about the involvement of lifeboat crews in medical emergencies at sea. The aim of this study is to analyze the medical operations at sea performed by the Royal Netherlands Sea Rescue Institution (KNRM).

**Methods:**

This is a retrospective descriptive analysis of all medical operations at sea performed by the KNRM between January 2017 and January 2020. The operations were divided in three groups: with ambulance crew aboard the lifeboat, ambulance crew on land waiting for the arrival of the lifeboat, and autonomous operations (without ambulance crew involvement). The main outcome measures were circumstances, encountered medical problems, follow-up and crew departure time.

**Results:**

The KNRM performed 282 medical operations, involving 361 persons. Operations with ambulance crew aboard the lifeboat (*n* = 39; 42 persons) consisted mainly of persons with serious trauma or injuries; 32 persons (76.2%) were transported to a hospital. Operations with ambulance crew on land (*n* = 153; 188 persons) mainly consisted of situations where time was essential, such as persons who were still in the water, with risk of drowning (*n* = 45, 23.9%), on-going resuscitations (*n* = 9, 4.8%) or suicide attempts (*n* = 7, 3.7%). 101 persons (53,7%) were transported to a hospital. All persons involved in the autonomous operations (*n* = 90; 131 persons) had minor injuries. 38 persons (29%) needed additional medical care, mainly for (suspected) fractures or stitches. In 115 (40.8%) of all operations lifeboat crews did not know that there was a medical problem at the time of departure. Crew departure time in operations with ambulance crew aboard the lifeboat (13.7 min, min. 0, max. 25, SD 5.74 min.) was significantly longer than in operations with ambulance crew on land (7.7 min, min. 0, max 21, SD 4.82 min., *p* < 0.001).

**Conclusion:**

This study provides new information about the large variety of medical emergencies at sea and the way that lifeboat and ambulance crews are involved. Crew departure time in operations with ambulance crew aboard the lifeboat was significantly longer than in operations with ambulance crew on land. This study may provide useful indications for improvement of future medical operations at sea, such as triage, because in 40.8% of operations, it was not known at the time of departure that there was a medical problem.

## Introduction

The Royal Netherlands Sea Rescue Institution (Koninklijke Nederlandse Redding Maatschappij—KNRM) has a significant role in Search and Rescue (SAR) operations in the Dutch part of the North Sea (58.000 km^2^) and most large open waters in the Netherlands [1]. KNRM lifeboat crews face a great variety of emergencies, from vessels with engine problems to medical emergencies. Lifeboat crews are trained to provide first aid, autonomously or supported by ambulance crew. The circumstances of these operations can be very difficult and possibly dangerous.

Very little data is available about the involvement of lifeboat crews in medical emergencies at sea [2–9]. The aim of this study is to become informed about medical emergencies at sea. Most of all, we were interested in understanding the differences between the ambulance supported medical operations and the autonomously performed operations by lifeboat crews.

### Setting

The KNRM has about 900 professionally trained volunteer lifeboat crew members**,** distributed over 45 lifeboat stations, who are available for SAR tasks. Depending on the type and size of the boat, 3–6 crew members have to be aboard a lifeboat before the boat can depart. At each station and at any time of the year, there are sufficient lifeboat crews available to depart. When an ambulance crew is on board, this team always consists of two persons, a nurse and a driver. Each of the 78 rescue boats is equipped with first aid material, while larger boats (*n* = 54) are also equipped with oxygen and an Automated External Defibrillator (AED). About 90% of all crew members have a valid Basic Life Support (BLS) and first aid certificate. Additional courses for all crew members address drowning, hypothermia, cervical spine injuries and the use of oxygen. Accommodated protocols, based on the relevant protocols from the national ambulance service, are aboard for consultation. The KNRM Radio Medical Service (Radio Medische Dienst – RMD) is available for consultation via very high frequency radio or mobile phone.

Lifeboat crew have been trained to observe and assess the health status as long as the person is aboard the lifeboat. This allows them in almost all situations to decide that no treatment, simple first aid treatment or transport to a local physician or hospital is needed. When in doubt, lifeboat crew contact the local physician or the KNRM Radio Medical Service that can be contacted 24/7 via Very High Frequency (VHF) or phone.

All lifeboat operations are coordinated by the Joint Rescue Coordination Centre (JRCC) of the Netherlands Coastguard. The JRCC receives direct emergency calls by telephone or VHF radio from victims or bystanders at sea or on other open waters. The JRCC also receives indirect incoming calls from regional dispatch centres when victims or bystanders on land call the national emergency telephone number 112. The JRCC decides if, which and how many lifeboats will take part in the operation and sent out the alarm to each of the available lifeboat crews of the rescue stations to become involved. The JRCC also decides if the deployment of an ambulance is deemed to be necessary. If so, the JRCC contacts the indicated Regional Ambulance Dispatch Centre. A protocol decides if the call meets the criteria to dispatch an ambulance from the involved Regional Ambulance Services. Ambulance crew make a decision for on-site treatment or transport based on national protocols and training.

The decision by the JRCC to ask for the deployment of an ambulance can be made at the same time as the lifeboat crew is alarmed and is then based on the available information regarding, among others, the status of the vessel in distress, the status of the crew, the location of the vessel, the distance to the closest harbour, and the present and future weather conditions. The person taking the call at the JRCC has no medical training and is unable to provide pre-arrival instructions on first aid or BLS. Another scenario is that an ambulance is deployed later during an operation when lifeboat crew themselves requests an ambulance.

Currently, there are no protocols indicating situations when support by an ambulance crew is advised and when lifeboat crews work autonomously. In the three years of this study, the KNRM registered 7385 SAR operations.

## Materials and methods

### Study design

This is a retrospective descriptive analysis of a consecutive cohort of all medical operations at sea performed by the KNRM between January 2017 and January 2020.

### Data collection

**Fig. 1:**
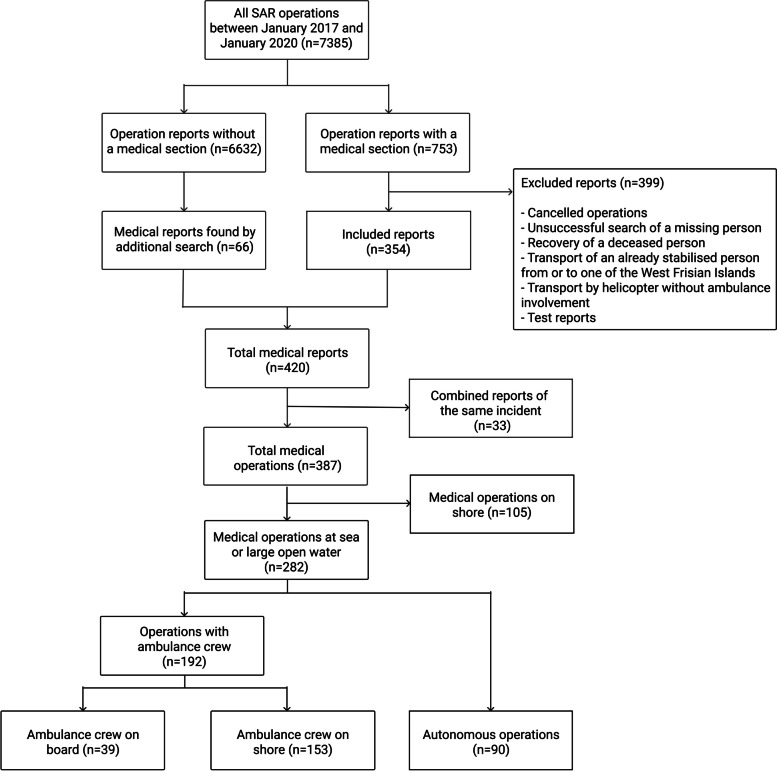
Prisma diagram describing the selection and exclusion of operations

After each operation the lifeboat’s captain fills out a structured online report, requiring general information about the operation (e.g., kind of operation, location, weather conditions). If a medical problem is part of the operation, an additionally available section about medical problems (divided into the main categories ‘trauma’, ‘illness’, ‘hypothermia’, ‘drowning’ and ‘suicide attempt’) and type of provided support has to be completed. All reports are collected in an internal online database (Internal registration system, KNRM, IJmuiden, The Netherlands) and double checked. All reports containing a completed medical section between January 2017 and January 2020 were included. An additional systematic search of the database was conducted to include missing reports. Reports that were not relevant for the objective of this study were excluded (Fig. 1). The selection and inclusion process were based on agreements between three persons (DM, TL, GP).

### Data processing

The data from the remaining rescue reports were automatically extracted and downloaded to a dedicated developed database. Missing data were obtained after contacting the lifeboat captain and were manually added. All data in the final database was completely anonymous and not traceable to individual persons, taking the general data protection regulation requirements into account. The complete set of data was double checked for incorrect input by two persons to assure data were collected accurately and without bias (DM, GP). All data was analyzed using IBM SPSS Statistics (Version 26.0 for Macintosh, 2019, Armonk, NY: IBM Corp.).

### Categories and definitions

Autonomous operations have been defined as all operations where lifeboat crews provided first aid based on training, existing protocols, and advice from the RMD, without ambulance crew involvement.

Medical problems have been categorized and defined as: trauma (persons with damage to skin, bones or organs, caused by any severity of trauma); illness (persons with cardiac, respiratory or other physical complaints); hypothermia (persons suffering from hypothermia or cold); drowning (persons with respiratory problems caused by submersion/immersion in fluid); suicide attempt (persons who made or attempted a suicide). When more than one medical problem is registered for a person, each problem is included in the database.

Crew departure time has been defined as the time between the moment the JRCC sends an alarm message to the individual crew members (standardized time on the pager) and their departure from the lifeboat station. All lifeboats are equipped with standardized clocks. It should be noted that there is always a delay between the moment the JRCC is alarmed and the moment the alarm message is send to the individual crewmembers. The amount of this delay was not part of this study and was therefore not registered. In operations where a first boat takes off without ambulance crew and the second boat takes off later for the same operation with ambulance crew on board, only the crew departure time of the first boat has been available because the registration system only registers crew departure time of the first lifeboat.

## Results

### Circumstances and population

Between January 2017 and January 2020, 282 medical operations were included. There was an annual increase in medical operations (2017: *n* = 73; 2018: *n* = 87; 2019: *n* = 122). Operations took place on large open waters connected to sea (*n* = 150, 53.3%), North Sea (*n* = 92, 32.6%), Wadden Sea (*n* = 32, 11.3%) or in a harbour (*n* = 8, 2.8%). The activity at the time of the operation was recreational boating (*n* = 122, 43.3%), commercial boating (*n* = 107, 37.9%) or other recreational activities, such as surfing or swimming (*n* = 53, 18.8%). Other characteristics can be found in Table 1.

**Table 1:**
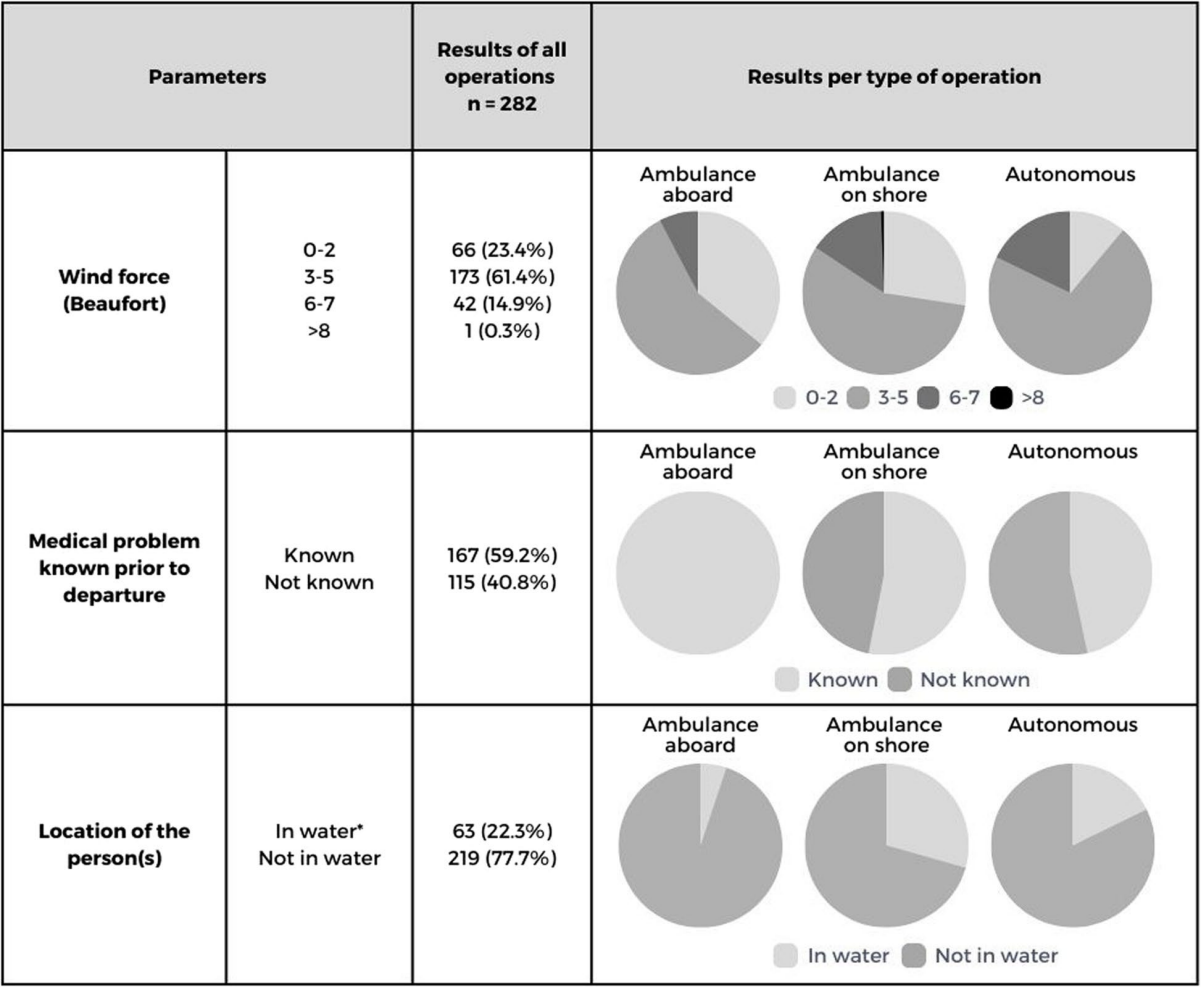
Circumstances of the operations

*Persons located in water were swimmers, surfers or passengers from a sunken or capsized vessel

In 192 operations ambulance crew supported lifeboat crews, either aboard the lifeboat (39 operations; 42 persons) or on land (153 operations;188 persons). In 90 operations (131 persons), lifeboat crews provided first aid to these persons autonomously, without involvement of ambulance crew.

Lifeboat crews provided first aid to 361 persons: 247 male (68.4%), 65 female (18%), 49 unknown (13.6%). The mean age was 38.8 years (*n* = 104, SD 19.7, min. 0, max 81). The three different groups (ambulance crew aboard, on land, autonomous) showed some differences in their profiles regarding circumstances (Table 1), medical problems (Table 2) and follow-up treatment once on land (Table 3).

**Table 2:**
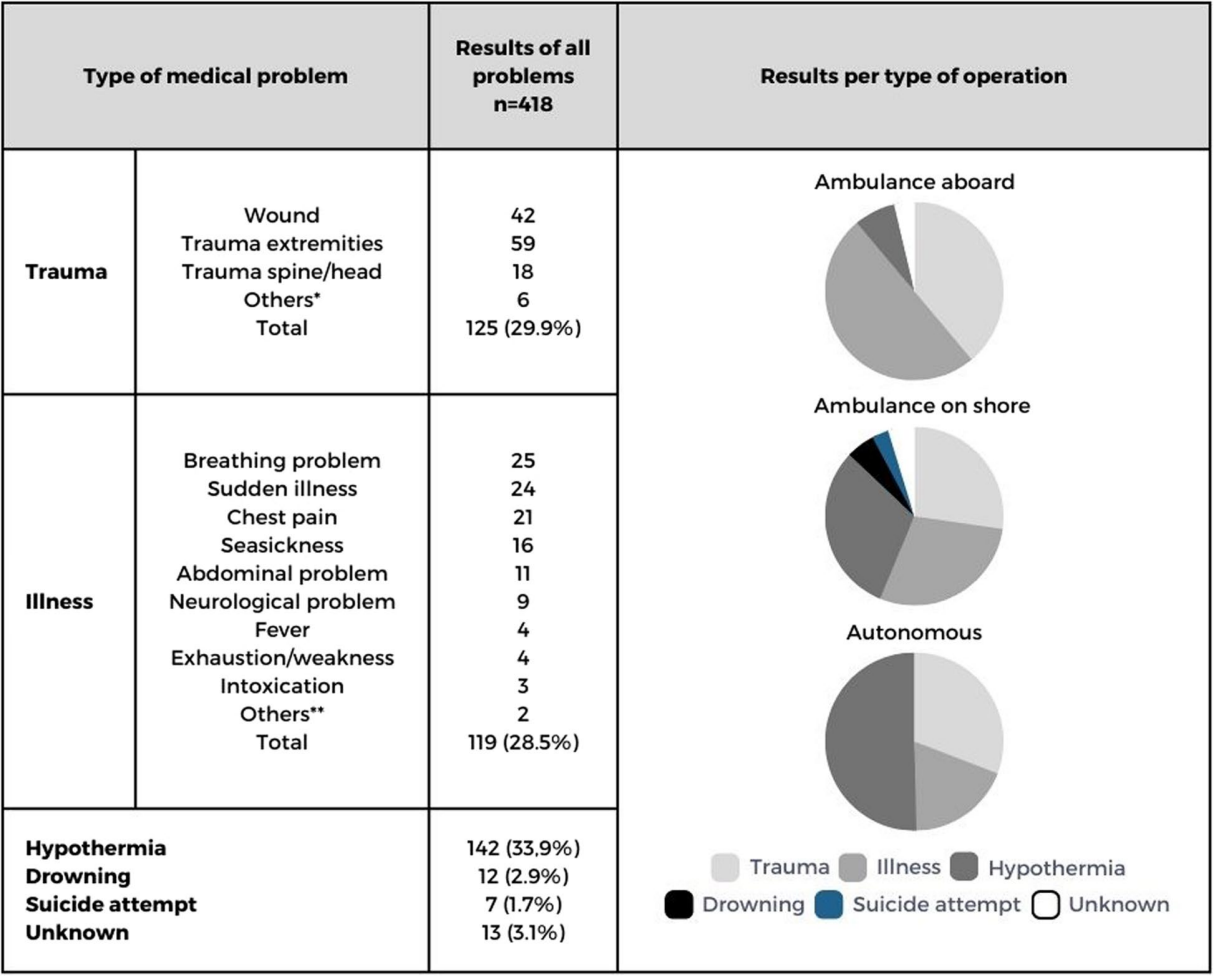
Medical problems

*Bite by insect/weeverfish (2x), nosebleed, locked jaw, testicular trauma, abdominal/thoracic trauma

**Thrombosis, hypoglycemia

### Ambulance crew aboard the lifeboat

The ambulance crew supported lifeboat crews in 42 persons aboard the lifeboat (11.6%). Prior to the departure, it was known in each of the operations, that a person with a medical problem was involved. Most of the persons suffered from trauma or illness (Table 2). These were often serious and ambulance crew transported 32 persons (76.2%) to a hospital. Six persons (14.3%) were treated at the scene (Table 3). For four persons (9.5%) the follow-up was unknown.

### Ambulance crew on land

In 188 persons (52.1%) the ambulance crew provided support as soon as lifeboat crews brought the person on land. Prior to the departure, it was known in 76 of the operations (49.7%), that a person with a medical problem was involved. In most of these operations there was an imminent life-threatening situation or the condition of the person could possibly deteriorate within a short amount of time, such as persons who were still in the water, with risk of drowning (*n* = 45, 23,9%), on-going BLS resuscitations (*n* = 9, 4.8%) or suicide attempt (*n* = 7, 3.7%) (Table 1 and Table 2). Once the boat reached land, 101 persons were transported to a hospital (53,7%); by ambulance (*n* = 98, 97%) or by private transport (*n* = 3, 3%). There were 63 persons (33.5%) treated at the scene. Four persons (2.1%) died at the scene, after resuscitation. For 20 persons (10.6%) the follow-up at the end of the operation was unknown (Table 3).

**Table 3:**
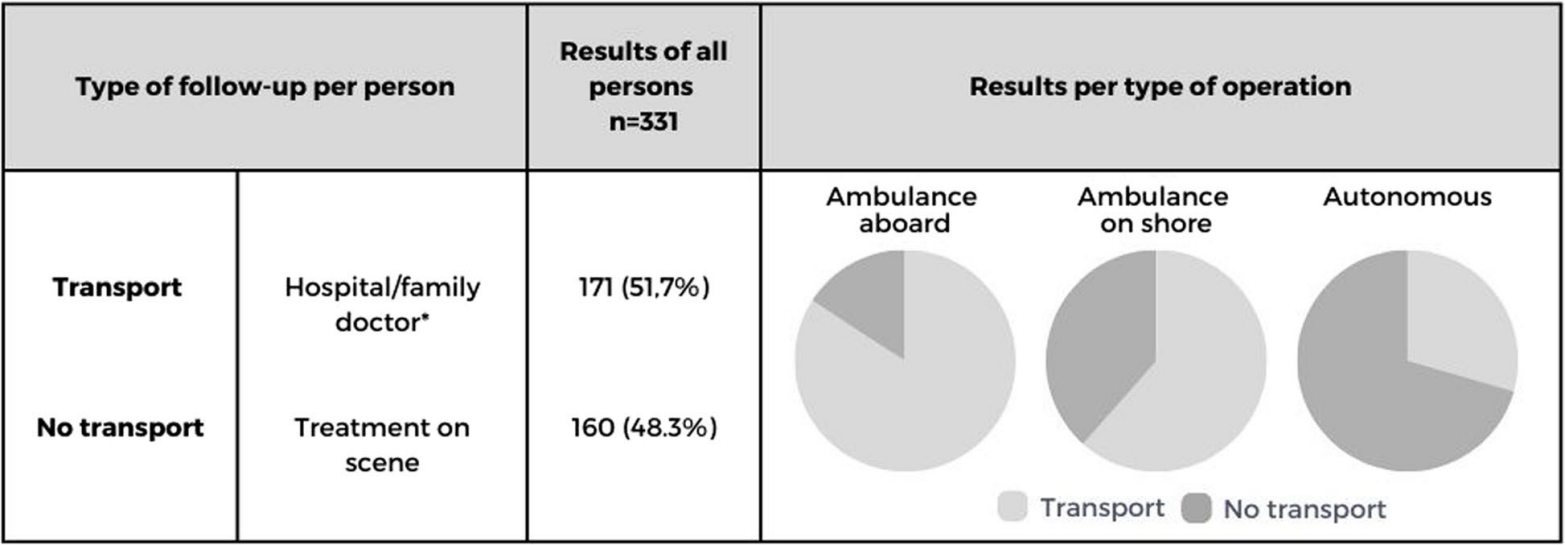
Follow-up of persons who were brought on land alive (*n* = 331, excluding 4 persons that died at the scene and 26 persons with unknown follow-up)

*All persons transported by ambulance were taken to a hospital

### Autonomous operations

Lifeboat crews provided first aid autonomously to 131 persons (36.3%), without involvement of ambulance crew. Prior to the departure, it was known in 48 of the operations (52.7%), that there was a person with a medical problem involved. All persons were conscious and had non-life-threatening medical problems, such as small wounds, stable fractures or seasickness. There were 67 persons (51.1%) suffering from hypothermia (Table 2). Lifeboat crews treated 91 (69.5%) persons at the scene. There were 38 persons (29%) who needed further medical follow-up for minor injuries. All of these persons were transported by private transport, either to a hospital or to a family doctor (Table 3). For two persons (1.5%) the follow-up at the end of the operation was unknown.

### Crew departure time

In the operations with ambulance crew aboard, there were two options: ambulance crew was aboard the first lifeboat or ambulance crew was aboard a second lifeboat, while the first lifeboat had immediately departed. The average crew departure time for operations with ambulance crew aboard the first lifeboat (*n* = 27, 1 crew departure time missing) was 13.7 min (min. 0, max. 25, SD 5.74 min, 95% CI 11.5–16.0). This was significantly longer than the crew departure time in operations with ambulance crew on land (*n* = 152, 1 crew departure time missing), which was 7.7 min (min. 0, max. 21, SD 4.82 min., 95% CI 6.9–8.5, *p* < 0.001) or with ambulance crew aboard the second lifeboat (*n* = 11, no crew departure time missing), which was 6.6 min (min. 0, max. 10, SD 2.54 min., 95%CI 4.8–8.3, *p* < 0.00). The average crew departure time in autonomous operations (*n* = 88, 2 crew departure times missing) was 8.2 min, significantly shorter than the crew departure time in operations with ambulance crew aboard (min. 0, max. 18, SD 5.41 min., 95% CI 7.0–9.4, *p* < 0.001).

## Discussion

This study showed that there is a large variety of medical emergencies at sea, while waiting for an ambulance crew significantly delays the crew departure time. Lifeboat crews assisted by ambulance crew aboard the lifeboat, predominantly treated persons with trauma or illness. 76% needed further treatment in hospital. In all these operations, lifeboat crews knew beforehand that a person with a medical problem was involved.

The operations with medical support by ambulance crew available on land were mainly acute situations (imminent risk of deterioration of the person’s condition or great distress at a vessel such as a fire or sinking). In the operations with a vessel in distress, lifeboat crews often found out after they arrived at the vessel that also a person with a medical problem was involved. 54% of all persons in this group needed further treatment in a hospital.

The majority of the autonomous operations concerned persons with non-urgent medical problems (such as mild hypothermia, small wounds, stable fractures or seasickness). KNRM lifeboat crews are trained for these types of medical problems and therefore able to provide first aid autonomously. In this group, 69,5% of the persons were treated on land without any need for further health consultancy. In case a physician’s consultancy was needed, this was often for a (suspected) fracture or a wound that needed stitches.

Few studies have reported about medical operations by lifeboat crews at sea. Studies on this subject endorse that these operations are diverse, demanding and complicated, caused by the combination of limited resources, large distance from land and medical facilities, and often serious medical problems [2, 3, 6, 7]. The studies also report a wide variety of medical problems, from small wounds to death, resembling the findings in this study [2, 3, 7, 10].

The majority of the problems were autonomously treated or treatment could be started by lifeboat crews until ambulance on land could take over. Almost always ambulance crew were requested for assistance on board when there was an appropriate indication. However, more than half of the persons that were initially treated on board without ambulance crew available needed medical consultancy once arrived on land. In 115 (40.8%) operations (Table 1), lifeboat crews did not know that there was a medical problem at the time of departure. More in-depth understanding is needed why the medical problems were not identified and to be able to improve the organization and the quality of the delivered medical support.

The decision to depart with or without ambulance crew had to be made based on minimal or no medical information, local knowledge and experience. Medical information is necessary to decide whether assistance by ambulance crew may be appropriate. Collecting this information is primarily a task of the JRCC, which dispatches the lifeboat crews and decides whether an ambulance crew on board is indicated. Studies show that emergency dispatch centres play an important role in improving the quality of pre-hospital care [11–15]. Furthermore, the use of standardized protocols results in faster identification, faster arrival at the scene and better recommendations to bystanders [16–18]. At this moment there are no clear protocols available describing in which situations lifeboat crews can depart for autonomous medical operations and when ambulance crew should be taken aboard the lifeboat. Development and implementation of protocols focusing on obtaining sufficient information about (possible) medical problems prior to dispatching lifeboat crews, could therefore attribute to better decisions about the need for ambulance support. Protocols could also be of benefit in providing uniformity across all rescue stations, ambulance services and the JRCC. The wide variety of medical problems and circumstances found in this study raise however concern if all situations can fit in protocols. On the spot decision making will often prevail. Furthermore, a program that allows the JRCC to advice on BLS and first aid measures, could attribute to support the persons in need of help before the arrival of lifeboat crews.

In addition, this is among the first studies to look into the involvement of ambulance crew in medical operations at sea. A study from Hawaii reports significant improvement in patient outcome after ambulance crew co-responded to emergencies at sea [4]. A German study demonstrated a higher severity of disease in maritime rescue compared to emergency medical services in a nearby town, recommending more professional support on board, for instance by an emergency physician [2]. One of the observations in this German study was the delay of departure (average of 18 min longer), due to the waiting time for arrival of the physician at the rescue station. This corresponds to the findings in our study, where the delay was less: 6 – 7 min. A possible explanation for the delay in our study is that ambulance services are active in a large area. This will often result in more time for an ambulance to reach the rescue station. Lifeboat crews are obliged to live or work within 10 min of the rescue station. It can however be questioned if this delay is clinically significant. For most of the medical problems in this category, it is unlikely that this delay increases morbidity or mortality.

Another option for future improvement would be to rely more on telecommunication, which provides a big potential for medical support in remote areas [19, 20].

### Limitations

This study was based on a database containing online reports, filled out by the lifeboat’s captain on a standardized form that has to be completed after each operation. The database has been assembled with great precision before the study had started, following a predetermined structure. With a few exceptions, all data was available. Nevertheless, there could be incorrect or missing information in some of the reports, leading to errors in the final database. Also, it is unknown whether the lifeboat’s captain used the standardized clocks on board or made an estimation of the crew departure time afterwards.

Transport times from the scene to land and the time of transfer to the ambulance are not registered. Also the transport times to hospital, registered by the ambulance services, are not available for this study. Pairing the information system from the KNRM with the ambulance information system could be an important step to assemble more and better information for future studies.

This study has a focus on the organization of medical first aid by lifeboat crews. The quality of the delivered care has not been the objective of this study. This is recognized as a relevant theme that needs further attention in the future.

This study uses the initial symptoms and diagnoses made by lifeboat crews. In most reports, the final diagnosis and outcome are not registered. This may have resulted in inaccuracy in the data base. At the same time, additional information gathered in the local and professional network will often have increased the accuracy of the symptoms and diagnosis.

The study has been limited to rather generic and operational data to obtain an overview of medical operations at sea by the KNRM, to understand what is happening in a cohort of three years, and to identify important topics for further studies. We believe that the study provides a first basis on which future in-depth studies can be build.

## Conclusion

This study analyzes the ambulance supported and autonomously performed medical operations by lifeboat crews at sea. It provides new information about medical operations at sea, showing that in the current practice (operations with ambulance crew aboard, ambulance crew on land and autonomous operations), each group has a characteristic profile in terms of medical problems and circumstances. Crew departure time in operations with ambulance crew aboard the lifeboat was significantly longer than in operations with ambulance crew on land. This study may provide useful indications for improvement of future medical operations at sea.

## Data Availability

By law and internal regulations, information from individual records is not allowed to leave the offices of the KNRM as this may result in the identification of individual persons. For this reason, the original datasets generated and analysed during the current study are not publicly available. The public available data sets (from 2017–2020) are available at https://www.knrm.nl/over-ons/wie-is-de-knrm/jaarverslagen-cijfers. Tables and figures that are at the basis of the current manuscript are available from the corresponding author on reasonable request.

## Abbreviations

AED: Automated External Defibrillator
BLS: Basic Life Support
JRCC: Joint Rescue Coordination Centre
KNRM: Koninklijke Nederlandse Redding Maatschappij (The Royal Netherlands Sea Rescue Institution)
RMD: Radio Medische Dienst (KNRM Radio Medical Service)
SAR: Search and Rescue
VHF: Very High Frequency
